# REDOX Imbalance and Oxidative Stress in the Intervertebral Disc: The Effect of Mechanical Stress and Cigarette Smoking on ER Stress and Mitochondrial Dysfunction

**DOI:** 10.3390/cells14080613

**Published:** 2025-04-19

**Authors:** Hui Li, Joshua Kelley, Yiqing Ye, Zhi-Wei Ye, Danyelle M. Townsend, Jie Zhang, Yongren Wu

**Affiliations:** 1 Department of Bioengineering, Clemson University, Charleston, SC 29425, USA; hui3@clemson.edu (H.L.); jkelle5@clemson.edu (J.K.); 2 Department of Orthopaedics and Physical Medicine & Rehabilitation, Medical University of South Carolina, Charleston, SC 29425, USA; 3 Academic Magnet High School, North Charleston, SC 29405, USA; 4 Department of Pharmacology and Immunology, Medical University of South Carolina, Charleston, SC 29425, USA; 5 Department of Drug Discovery and Biomedical Sciences, Medical University of South Carolina, Charleston, SC 29425, USA

**Keywords:** low back pain, intervertebral disc degeneration, environmental risk factors, reactive oxygen species, redox imbalance, endoplasmic reticulum stress, mitochondrial dysfunction, ER–mitochondrial crosstalk, cellular dysfunction

## Abstract

Low back pain is a widespread condition that significantly impacts quality of life, with intervertebral disc degeneration (IDD) being a major contributing factor. However, the underlying mechanisms of IDD remain poorly understood, necessitating further investigation. Environmental risk factors, such as mechanical stress and cigarette smoke, elevate reactive oxygen species levels from both endogenous and exogenous sources, leading to redox imbalance and oxidative stress. The endoplasmic reticulum (ER) and mitochondria, two key organelles responsible for protein folding and energy production, respectively, are particularly vulnerable to oxidative stress. Under oxidative stress conditions, ER stress and mitochondrial dysfunction occur, resulting in unfolded protein response activation, impaired biosynthetic processes, and disruptions in the tricarboxylic acid cycle and electron transport chain, ultimately compromising energy metabolism. Prolonged and excessive ER stress can further trigger apoptosis through ER–mitochondrial crosstalk. Given the unique microenvironment of the intervertebral disc (IVD)—characterized by hypoxia, glucose starvation, and region-specific cellular heterogeneity—the differential effects of environmental stressors on distinct IVD cell populations require further investigation. This review explores the potential mechanisms through which environmental risk factors alter IVD cell activities, contributing to IDD progression, and discusses future therapeutic strategies aimed at mitigating disc degeneration.

## 1. Introduction

Low back pain, which affects approximately 70% of adults at least once in their lifetime, significantly reduces quality of life and imposes a substantial economic burden on society [1,2]. One major contributor to low back pain is intervertebral disc degeneration (IDD), which is both a chronic and progressive condition [3]. Despite its prevalence, the molecular mechanisms underlying IDD remain poorly understood.

**Figure 1:**
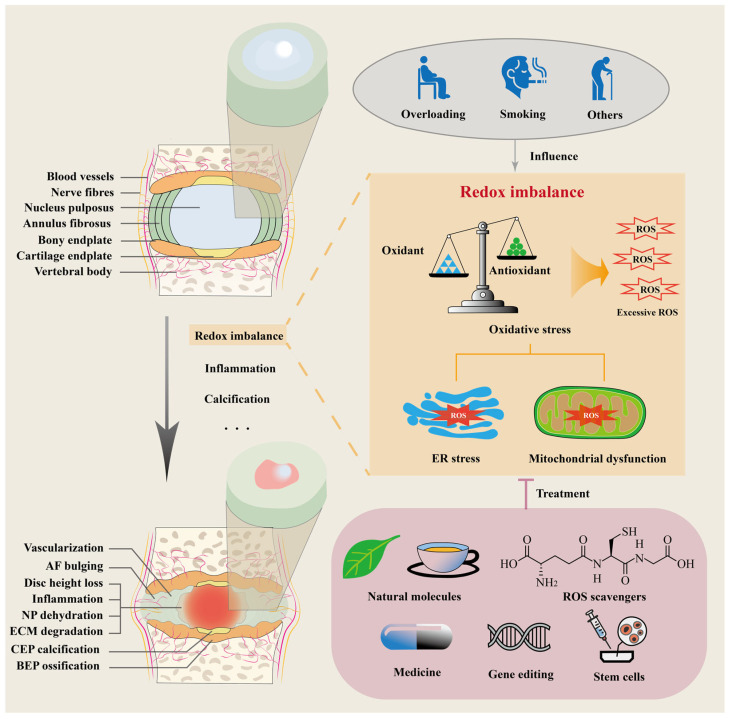
Comparison of healthy and degenerative intervertebral disc (IVD): redox imbalance may be a potential contributor. Morphological and compositional changes in intervertebral disc degeneration (IDD) are characterized by nucleus pulposus (NP) dehydration, annulus fibrosus (AF) bulging, cartilage endplate (CEP) calcification, bony endplate ossification, and disc height reduction, along with extracellular matrix (ECM) remodeling, vascularization, and inflammation. One potential contributor to these changes is redox imbalance. Various environmental risk factors disrupt redox homeostasis in IVD cells, leading to excessive reactive oxygen species (ROS) production, which induces endoplasmic reticulum (ER) stress and mitochondrial dysfunction, ultimately altering cellular activity. Therapeutic strategies aim to scavenge excessive ROS, alleviate ER stress, and restore mitochondrial function, thereby mitigating or slowing the progression of IDD.

The intervertebral disc (IVD) consists of three primary components: the nucleus pulposus (NP), annulus fibrosus (AF), and endplate, which includes both the bony endplate and the cartilage endplate (CEP) (Figure 1) [4,5]. The NP, a gelatinous structure in the center of the IVD, is composed of 70–90% water, collagen II, proteoglycans, and non-collagenous proteins [6]. Encircling the NP, the AF forms fibrous rings of concentric lamellae [7,8], each containing highly organized collagen I and II fiber bundles [6]. The CEP, primarily composed of hyaline cartilage rich in collagen II, proteoglycans, and water, serves as an important mechanical and transport interface, connecting the NP and AF to the vertebral body [9,10]. This structural composition enables the avascular IVD to perform load-bearing and shock-absorbing functions while also facilitating nutrient transport to the NP and inner AF cells from surrounding tissues [9]. Specialized cell types—including notochordal-derived cells [11], fibroblasts and fibrochondrocytes [12], and chondrocytes [9]—in the NP, AF, and CEP, respectively, are responsible for maintaining their localized extracellular environment. Dysfunction in these cells can initiate IDD, leading to both morphological and compositional alterations within the IVD (Figure 1) [5,13,14,15,16,17]. These changes include reduced proteoglycan content and loss of hydration in the NP [5,14], AF bulging and reduced glycosaminoglycan content [15,16], and CEP thinning and calcification [17]. Altered cellular behavior, marked by dysregulated anabolic and catabolic activities, plays a key role in IDD development and can be influenced by environmental factors such as abnormal mechanical stress, cigarette smoking, and other conditions, which accelerate its progression [4,18,19,20]. A critical mechanism linking these environmental factors to IVD degeneration is the disruption of cellular reduction–oxidation (redox) homeostasis.

**Figure 2:**
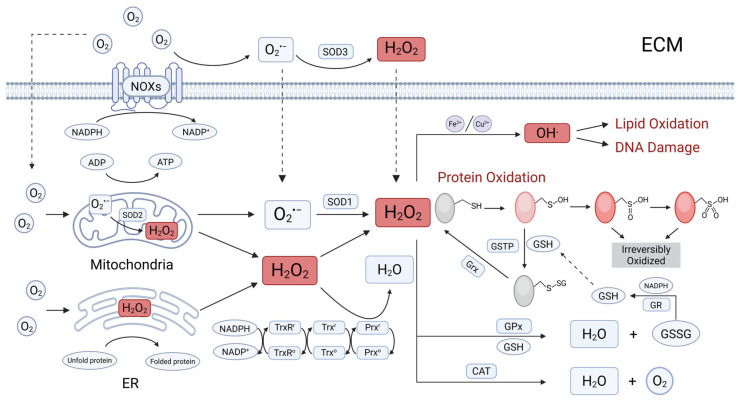
Cellular ROS generation and antioxidant defense mechanisms. Cellular ROS, including radical and non-radical oxygen species, primarily originate from three key sites: the NADPH oxidase (NOX) complex, the ER, and mitochondria. Redox homeostasis is maintained through a balance between pro-oxidant and antioxidant processes. ROS are neutralized by detoxifying enzymes such as superoxide dismutase (SOD), catalase (CAT), glutathione peroxidase (GPx), peroxiredoxin (Prx), and thioredoxin (Trx). NADPH and glutathione (GSH) serve as major reducing agents, supporting antioxidant enzymes like glutathione reductase (GR), thioredoxin reductase (TrxR), and GPx. Additionally, post-translational modifications such as S-glutathionylation and deglutathionylation, mediated by glutathione S-transferase P (GSTP) and glutaredoxin (Grx), regulate protein function under oxidative and nitrosative stress. Excessive ROS production can overwhelm antioxidant defenses, leading to cellular damage, including protein and lipid oxidation as well as DNA damage. Red indicates ROS production and its associated molecular damage.

Redox dynamics involve electron transfer in redox reactions, where one chemical species undergoes reduction while another undergoes oxidation [5]. Redox homeostasis, the balance between the production and elimination of reactive oxygen species (ROS) and reactive nitrogen species (RNS), is essential for executing various cellular processes, including signal transduction, metabolic regulation, mitochondrial bioenergetics, protein folding, and cell fate determination [21,22,23]. ROS are chemically reactive molecules derived from oxygen, while RNS arise from both nitrogen and oxygen. RNS typically have longer half-lives than ROS but are less abundant. Major ROS include superoxide anions (O_2_^•−^), hydrogen peroxide (H_2_O_2_), which serves as an important signaling molecule, and hydroxyl radicals (OH•), which are highly cytotoxic [24,25,26]. The primary sources of ROS production are the mitochondrial electron transport chain (ETC), the endoplasmic reticulum (ER), and the NADPH oxidase (NOX) complex (Figure 2) [23]. Mitochondria, especially complexes I and III of the ETC, are significant sites of O_2_^•−^ generation, with their activity modulated by membrane potential and oxygen availability. The ER also contributes to ROS production during oxidative stress, primarily through protein folding processes involving enzymes like protein disulfide isomerase (PDI) and endoplasmic reticulum oxidoreductase 1 (ERO1). Additionally, ER stress-induced calcium (Ca^2+^) leakage into the cytosol can exacerbate O_2_^•−^ and H_2_O_2_ generation. NOX enzymes, the only enzymes primarily responsible for generating ROS, transfer electrons from NADPH to molecular oxygen, resulting in O_2_^•−^ formation. H_2_O_2_ is mainly produced as a byproduct of enzymatic reactions, particularly through superoxide dismutase (SOD), which converts O_2_^•−^ into H_2_O_2_ and O_2_. OH• are generated when O_2_^•−^ or H_2_O_2_ interact with metal ions such as iron (Fe^2+^) or copper (Cu^2+^), facilitating the Fenton reaction (involving Fe^2+^) or Fenton-like reactions (involving Cu^2+^), which results in the formation of highly reactive OH•. Other enzymes, including xanthine oxidase, lipoxygenase, and cyclooxygenase (COX), also contribute to ROS production, with their activity varying depending on specific cellular and tissue contexts. Given their pivotal role and increased abundance relative to RNS, understanding ROS and the mechanisms which regulate it is crucial for understanding its impact on cellular health.

Cellular ROS levels are tightly regulated by endogenous antioxidant systems to maintain homeostasis, which include both enzymatic and non-enzymatic defenses [23]. Key antioxidants, such as vitamins C and E, glutathione (GSH), and antioxidant enzymes like SOD, catalase, glutathione peroxidases (GPx), peroxiredoxins (Prx), thioredoxin (Trx), glutathione reductase and thioredoxin reductase (TrxR), and sulfiredoxin (Srx) play crucial roles in ROS neutralization. SOD catalyzes the dismutation of O_2_^•−^ into H_2_O_2_, which is then broken down into water and oxygen by catalase. GPx reduces peroxides using GSH as a cofactor, while Prx and Trx reduce peroxides through thiol-disulfide exchange. NADPH is essential for recycling oxidized GSH and oxidized Trx through glutathione reductase and TrxR, respectively. Srx further supports antioxidant defense by restoring overoxidized Prx [27]. Additionally, FADH_2_, primarily generated in the tricarboxylic acid (TCA) cycle and ETC, donates electrons to respiratory complexes, indirectly regulating ROS levels by sustaining mitochondrial function and oxidative phosphorylation. Selenium also plays a key role in antioxidant defense, as selenoenzymes provide enhanced resistance to oxidation compared to sulfur-containing enzymes. Beyond enzymatic activity, post-translational modifications such as S-glutathionylation and deglutathionylation, mediated by glutathione S-transferase and glutaredoxin (Grx), respectively, modify proteins in response to oxidative and nitrosative stress, influencing cellular functions [28]. Complementing these defenses, the transcription factor Nrf2 (nuclear factor erythroid 2-related factor 2) serves as a master regulator of antioxidant responses. Under oxidative stress, Nrf2 translocates to the nucleus and activates the expression of various antioxidant genes, further bolstering the cell’s defense mechanisms [29]. However, environmental factors can disrupt this homeostasis leading to unchecked ROS generation and subsequent oxidative stress [5,18]. This stress can cause irreversible damage to biomolecules such as proteins, DNA, and lipids, resulting in senescence and cell death [30], which collectively contribute to cellular, tissue, and organ damage, impacting systemic health.

Oxidative stress affects cellular organelles, such as the ER and mitochondria [5,31]. In the ER, it exacerbates protein misfolding and unfolding, while also disrupting Ca^2+^ homeostasis, leading to ER stress [31,32]. In mitochondria, oxidative damage impairs mitochondrial biogenesis and disrupts membrane potential, leading to mitochondrial dysfunction and intracellular signaling [5,33]. Collectively, these dysfunctions contribute to reduced biosynthesis, cellular apoptosis, inflammation, and extracellular matrix (ECM) degradation, ultimately accelerating the progression of IDD [5,31,33].

The effect of environmental risk factors on redox homeostasis contributes to oxidative stress, which has systemic implications across various organ systems, including the respiratory, cardiovascular, and musculoskeletal systems. This review focuses on the impact of these mechanisms in IDD, offering insights into their role in IDD pathogenesis and highlighting potential therapeutic strategies to mitigate disease progression. Additionally, while varying levels of oxidative stress and duration of the environmental risk factors determine how cells will respond, this review will specifically focus on the apoptotic response as oxidative-stress-induced cellular senescence in the IVD has already been covered in previous literature reviews [5,34,35].

To perform this review, PubMed, Medline, and SCOPUS were searched using various combinations of the following keywords: cigarette smoking, mechanical loading, intervertebral disc, annulus fibrosus, nucleus pulposus, cartilage endplate, mitochondria, endoplasmic reticulum, oxidative protein folding, cell signaling, oxidative stress, reactive oxygen species, and redox homeostasis. Articles were excluded if they employed inappropriate methodologies, did not focus on the above-mentioned topic, or were published prior to 1990.

## 2. Environmental Risk Factors Leading to Redox Imbalance

The extracellular microenvironment plays a crucial role in regulating cellular activities through intra- and inter-cellular communication [36]. Environmental factors can create adverse conditions in the microenvironment that induce excessive ROS generation and impair antioxidant defense systems by introducing exogenous substances and modulating signaling pathways (Figure 2). While there are various environmental stressors that can alter the IVD microenvironment, the adverse conditions created by mechanical stress and cigarette smoking will be examined in this review [4,18].

### 2.1. Mechanical Stress

**Figure 3:**
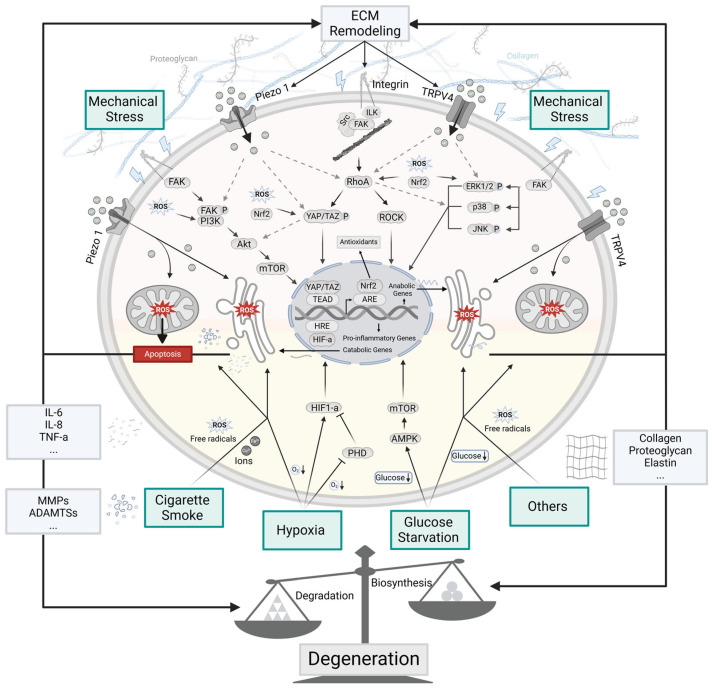
The mechanism through which environmental risk factors contribute to IDD. Environmental risk factors, whether through the introduction of exogenous substances—such as free radicals and heavy ions from cigarettes and other environmental sources—or the modulation of key signaling pathways triggered by mechanical stress, ROS, antioxidants, low oxygen, and low glucose concentrations, contribute to excessive ROS generation and the impairment of the antioxidant defense system. The accumulation of excessive ROS in IVD cells induces oxidative stress, particularly affecting the ER and mitochondria, leading to ER stress and mitochondrial dysfunction. These alterations disrupt protein synthesis and modification as well as cellular energy supply, ultimately disturbing the balance between biosynthesis and degradation. Consequently, this imbalance drives ECM remodeling by impairing biosynthesis, increasing cell apoptosis, promoting the release of inflammatory factors, and accelerating ECM degradation.

IVD cells experience various mechanical forces, and the magnitude, frequency, mode, and duration of these forces influence cellular activity [37]. Cells respond to mechanical stimuli using mechanosensitive ion channels [38,39], membrane proteins [40], and cytoskeletal components [41]. Under healthy loading conditions, IVD cellular metabolic processes proceed without altering tissue structure or compromising its mechanical properties [42]. However, excessive or high-frequency loadings can induce mechanical stress influencing cell fate, triggering inflammation responses, degrading the ECM, and ultimately altering tissue structure [13,43]. Mechanical stressors can also influence redox homeostasis by activating mechanosensitive ion channels, such as Piezo1 and transient receptor potential cation channel subfamily V member 4 (TRPV4). These channels increase Ca^2+^ influx, which can promote excessive ROS generation and trigger downstream mechanotransduction pathways (Figure 3) [44]. Additionally, mechanical stress stimulates mechanotransduction receptors, activating focal adhesion kinase (FAK), a central hub for multiple downstream signaling pathways including PI3K/Akt, RhoA/ROCK, YAP/TAZ, and MAPK [45,46]. FAK is a tyrosine kinase that influences migration and cellular contacts. Its activity is regulated by phosphatases which undergo S-glutathionylation reactions following ROS signaling [47]. Collectively, these pathways regulate antioxidant levels and cellular processes, which, in turn, feedback to modulate the pathways themselves [4,48]. However, excessive ROS can inhibit these pathways, impairing antioxidant defense system [49,50].

#### 2.1.1. Piezo 1 Channel

The Piezo 1 channel is responsible for ion transport into the cell cytoplasm and exhibits a slight selectivity for Ca^2+^ (Figure 3) [51,52]. Mechanical stress can activate Piezo1, leading to Ca^2+^ influx into cells and inducing excessive ROS generation [38]. One study examining human NP tissue found that as ECM stiffness increased, Piezo1 was upregulated, accompanied by an increase in cytosolic Ca^2+^ and intracellular ROS. This finding establishes a link between Piezo1 activation, Ca^2+^ concentration and ROS generation [38]. Notably, Piezo1 knockdown has been shown to attenuate these effects [38]. Some studies suggest that the Piezo1 channel plays a role in antioxidant expression, as evidenced by its upregulation coinciding with Nrf2 activation [53,54], potentially through interactions with downstream pathways [55,56,57]. However, the relationship between Piezo1 and antioxidant expression in IVD requires additional investigation.

The influx of Ca^2+^ also initiates a cascade of cellular responses, including biosynthesis, apoptosis, inflammation, and degradation. In injured or degenerative IVDs, Piezo1 activation is associated with reduced collagen II and aggrecan gene expression [58,59]. In AF cells, excessive mechanical stress can induce apoptosis via the Ca^2+^/Calpain2/Bcl-2-associated X protein (Bax)/caspase (Cas)-3 pathway [39], while in NP cells, it upregulates Bax, p53, p16, and p62 and downregulates B-cell lymphoma 2 (Bcl-2) and LC3-I/LC3-II [58]. Additional studies have shown increased Piezo1 activity and upregulated inflammatory factors (Tumor Necrosis Factor-α (TNF-α), Interleukin (IL)-6, IL-1β, and NLRP3) and catabolic markers (Matrix Metalloproteinase (MMP)-1 and MMP-9) when mechanical stresses were present [58,59,60]. Moreover, given that CEP chondrocytes exhibit mechanosensitive capabilities [61], investigation of Piezo1 signaling in CEP cell populations is necessary.

#### 2.1.2. TRPV4 Channel

TRPV4 is another nonselective ion channel that contributes to redox signaling and mechanotransduction (Figure 3). Under normal physiology, it is involved in regulating systemic osmotic pressure that is necessary for the structural integrity of the cytoskeleton. The activation of TRPV4 induces ROS generation, with studies showing that TRPV4-mediated Ca^2+^ influx plays a key role in mitochondrial ROS release, contributing to redox regulation [62]. Additionally, TRPV4 is implicated in antioxidant regulation, potentially through interactions with downstream pathways [63,64,65,66]. For example, TRPV4 inhibition has been shown to enhance the activity of antioxidative enzymes SOD, GPx, and catalase via the Akt/Nrf2/ARE pathway [63]. However, its specific role in IVD cells remains to be further investigated.

TRPV4 also contributes to the regulation of essential cellular processes. In TRPV4 knockdown mice, proteoglycan protein levels were lower in the AF compared to those in wild-type mice; however, the gene expression of aggrecan, biglycan, and proteoglycan 4 was higher in the knockdown mice [67]. Furthermore, TRPV4 activation promotes inflammation by upregulating IL-6, IL-8, and COX-2 [64,65,68]. Collectively, these findings underscore the role of TRPV4 in cellular homeostasis.

#### 2.1.3. PI3K/Akt Signaling Pathway

Phosphoinositide-3-kinases (PI3Ks) regulate growth and motility through generating lipid second messengers. Among those pathways activated are the Nrf2-mediated antioxidant defense system. The PI3K/Akt signaling pathway is responsible for enhancing antioxidant defense by increasing antioxidant enzyme production (Figure 3). Upon activation, PI3K promotes Akt signaling, which subsequently increases Nrf2 and Heme oxygenase-1 (HO-1) expression [69,70], leading to increased synthesis of antioxidant enzymes such as SOD, which helps mitigate oxidative stress [49]. However, how antioxidants regulate the PI3K/Akt pathway remains unclear. Additionally, excessive ROS can inhibit PI3K phosphorylation in NP cells [49], suggesting that oxidative stress may impair this protective pathway.

Beyond antioxidant regulation, the PI3K/Akt pathway plays a crucial role in IVD cell survival, influencing biosynthesis, apoptosis, and ECM degradation. PI3K/AKT signaling can enhance mechanistic target of rapamycin (mTOR) expression, promoting protein synthesis and cell proliferation [70,71]. Activation of PI3K/Akt by N-acetylserotonin has been shown to promote collagen II and aggrecan synthesis in NP cells exposed to H_2_O_2_ [49]. Similarly, other studies have also reported an increase in collagen II gene (Col2A1) expression following the activation of this pathway [72,73]. Furthermore, this pathway protects IVD cells from apoptosis by upregulating Bcl-2 and Bcl-xL (anti-apoptotic proteins) while downregulating Bax and Bak (pro-apoptotic proteins). This inhibits Cas-9 and Cas-3 levels, suppressing apoptosis [49,72]. PI3K/Akt activation has also been shown to downregulate matrix metalloproteinases (MMP-1, MMP-3, and MMP-13) in human NP cells, influencing ECM degradation [49,74].

Overall, the PI3K/Akt pathway plays a multifaceted role in IVD health by maintaining redox balance, promoting biosynthesis, and preventing apoptosis. However, the mechanisms though which this pathway specifically regulates redox balance and other cell functions in different regions of the IVD, particularly the AF and CEP, remain to be elucidated.

#### 2.1.4. RhoA/ROCK Signaling Pathway

Rho-associated protein kinase or Rho-associated coiled-coil kinase (RhoA/ROCK) is a serine threonine kinase that contributes to antioxidant regulation (Figure 3) while also influencing cell survivability through its effects on the cytoskeleton. Studies suggest that Nrf2 enhances cell motility by upregulating the RhoA/ROCK pathway, while Nrf2 overexpression or inhibition produces opposite effects, indicating its potential involvement in redox balance [75]. However, the specific role of the RhoA/ROCK pathway in regulating the antioxidant system in IVD cells remains unclear.

The RhoA/ROCK pathway also regulates cell survivability [76]. Mechanical stretch has been shown to activate this pathway, leading to the release of pro-senescence markers such as p16 and p53 in rat AF cells [77]. NOX 4-derived ROS can downregulate the RhoA/ROCK pathway, leading to reduced cell proliferation [78]. Additionally, the RhoA/ROCK pathway also exhibits extensive crosstalk with other signaling cascades, such as the MAPK pathway, to regulate various cellular activities [79,80].

#### 2.1.5. YAP/TAZ Signaling Pathway

Yes-associated protein (YAP/TAZ) functions as a sensor of the cells’ physical structure, shape and polarity and is crucial for redox regulation (Figure 3). Upon activation, YAP promotes transcription of Forkhead Box M1(FOXM1), which subsequently enhances Nrf2-mediated GSH synthesis, linking YAP/TAZ to antioxidant production [81,82]. YAP knockdown mice show reduced FOXM1, Nrf2, and HO-1 expression, along with increased ROS accumulation and apoptosis [81]. In turn, Nrf2-induced antioxidants can activate YAP, creating a positive feedback loop that enhances antioxidant production [82,83]. Additionally, ROS can influence YAP/TAZ signaling by promoting the phosphorylation of Mst1/2 and Lats1/2, which inhibits YAP/TAZ activity and impairs upregulation of antioxidants [50].

The YAP/TAZ pathway also plays a crucial role in regulating biosynthesis, apoptosis, and degradation [84,85]. High-magnitude hydrostatic pressure can enhance Hippo-YAP/TAZ signaling, leading to decreased levels of collagen II, aggrecan, and sulfated glycosaminoglycan content [86]. It can also induce a cell apoptotic response through the upregulation of Cas-3 [86]. Moreover, dynamic loading activates the Hippo-YAP/TAZ pathway in NP cells, upregulating catabolic markers, including the gene expression of ADAMTS4 and MMP-13 [87]. The YAP/TAZ pathway also interacts with other signaling pathways, such as PI3K/AKT, to maintain cellular homeostasis [84].

Overall, the YAP/TAZ pathway plays a significant role in IVD cellular activities regulation. However, the specific interaction between YAP and Nrf2 in IVD cell populations, particularly in AF and CEP cells, requires further investigation.

#### 2.1.6. MAPK Signaling Pathway

Mitogen-activated protein kinases (MAPKs) initiate signaling pathways in response to a wide variety of stimuli, including cytokines, heat shock and osmotic stress. The MAPK signaling can initiate three primary cascades, ERK1/2, p38 MAPK, and JNK, each of which can play a role in redox regulation (Figure 3). Under non-stressed conditions, JNK forms protein–protein interactions with glutathione S-transferase P (GSTP) where it serves as an inhibitor [88]. In GSTP-null mice, higher constitutive levels of JNK activity have been detected. Oxidative stress and ultraviolet radiation led to the dissociation of this complex and the activation of these pathways. In separate studies, it was shown that apoptosis in NP cells under static mechanical pressure was alleviated through activation of the MAPK/Nrf2/HO-1 pathway, leading to reduced ROS levels [89]. This may be attributed to its role in promoting Nrf2-mediated antioxidant synthesis like GSH, enhancing antioxidant protection [89,90]. Additionally, another study reported that p38 MAPK inhibition reduced Nrf2 activation [90]. In turn, Nrf2 can suppress p38 MAPK activation to mitigate inflammation under low-frequency tensile stress [48]. However, how antioxidants interact with the MAPK pathway to enhance defense remains unclear. Moreover, ROS can inhibit ERK and JNK activation [91], potentially impairing the antioxidant defense system.

The MAPK pathway also plays an essential role in regulating biosynthesis, senescence, and inflammation [92]. Cyclic tensile stress has been shown to activate MAPK pathways, including ERK, SAPK/JNK, and p38-MAPK, in AF cells; however, no significant ECM turnover was observed in the cell culture conditions [93]. In contrast, high-magnitude compression activated the p38 MAPK-ROS pathway, leading to decreased levels of collagen II, aggrecan, and glycosaminoglycan content [94]. Additionally, matrix stiffness has been implicated in regulating NP cell activities, potentially through the integrin β1–p38 MAPK pathway [95]. Moreover, the MAPK pathway also modulates the release of inflammatory factors such as IL-6, IL-8, and COX-2 [93,96].

### 2.2. Cigarette Smoking

Cigarette smoking exacerbates the microenvironment of IVD cells through both direct mechanisms, such as the introduction of toxic components into the microenvironment [97,98], and indirect mechanisms, such as the limitation of nutrient availability (Figure 3). Cigarette smoke limits nutrient availability as carbon monoxide and nitric oxide, constituents of cigarette smoke, bind to hemoglobin more readily than oxygen, thereby reducing its oxygen saturation [99]. Additionally, it induces vasoconstriction, which impairs blood supply to the IVD [100], further restricting nutrient availability, exacerbating hypoxia and glucose starvation [101,102]. Recent studies also suggest that endplate remodeling contributes to reduced nutrient diffusion, further compromising the IVD microenvironment when smoke exposure is involved [17,103].

#### 2.2.1. Cigarette Smoke

Cigarette smoke introduces ROS from three primary sources: (1) exogenous ROS that are directly present in the smoke; (2) ROS generated through the interaction of its constituents with cellular components; and (3) endogenous ROS production triggered by cigarette smoke. First, cigarette smoke comprises over 7000 chemical compounds [104], and among these are free radicals and ROS [105]. Certain long-lived radical species, such as o- and p-benzosemiquinone, are considered secondary oxidative byproducts [106,107], and may act as exogenous ROS. However, due to their transient nature, the toxicological impact and bioavailability of these radicals in the human body remains to be elucidated. Secondly, redox-active constituents in cigarette smoke can participate in ROS generation. For example, Benzosemiquinones can penetrate the air–blood barrier in the lungs, entering the bloodstream where they can interact with hemoglobin and albumin to produce O_2_^•−^ [108,109,110]. Additionally, transition metals in cigarette smoke like Fe^2+^ generate ROS due to their variable oxidation states [111,112]. Fe^2+^ overload induces oxidative stress and ferroptosis, contributing to ECM degradation and mitochondrial dysfunction, while Fe^2+^ chelation mitigates these effects [112]. Moreover, Fe^2+^ homeostasis, regulated by glutaminase 1 (GLS1) and cysteine desulfurase (NFS1), is essential for preventing ferroptosis and senescence in NP cells [113]. This suggests that heavy metal could be a source of ROS generation. Thirdly, cigarette smoke interferes with various cellular metabolic processes. Studies have shown that cigarette smoke activates NOX [114,115], with cellular Src (c-Src) serving as a mediator for this activation [116,117], leading to increased O_2_^•−^ production [114]. Additionally, cigarette smoke disrupts the mitochondrial ETC, particularly at Complex I and II [118], resulting in accelerated electron leakage and O_2_^•−^ formation [118,119]. Additionally, cigarette smoke can compromise antioxidant systems by disrupting the balance between antioxidant consumption and synthesis [120,121]. The effects of cigarette smoke on metabolic processes and antioxidant defense in IVD cells require further investigation.

#### 2.2.2. Hypoxia

Cigarette smoking indirectly causes a significant drop in oxygen levels (hypoxia) by reducing the hemoglobin oxygen saturation and inducing vasoconstriction. Hypoxia is associated with elevated levels of ROS. Various cell types, including brain and endothelial cells, have been used to show enhanced ROS production under hypoxic conditions [122,123]. For instance, endothelial and brain cells exhibit increased O_2_^•−^ production under acute hypoxia [123]. To restore homeostasis, cells orchestrate a coordinated response that involves the activation of a transcription factor, hypoxia inducible factor (HIF) [124], as well as NF-kB. In fact, it has been shown that elevated ROS leads to S-glutathionylation of subunits of NF-kB, which, in turn, alters their binding to DNA and ultimately negatively regulates antioxidant responses [125,126]. Hypoxia-induced increases in ROS also impact mitochondrial bioenergetics. A major source of ROS includes electron leakage from mitochondrial ETC complexes, especially complex III [122,127], with complexes I and II contributing as well [128,129,130]. Additionally, enzymatic sources, such as NOXs, and activation of the HIF-1α pathway also contribute to ROS generation under hypoxic conditions [131,132].

Under normoxic conditions, HIF-1α degradation is facilitated Von Hippel-Lindau via prolyl hydroxylase domain enzymes [133]. Conversely, under hypoxia, this enzyme activity is inhibited, resulting in HIF-1α stabilization and accumulation, subsequently activating the transcription of genes regulating energy homeostasis, glucose utilization, and angiogenesis [132,134,135]. In contrast, some studies have reported decreased ROS generation under hypoxic conditions, with studies showing that human embryonic kidney cells under acute hypoxia exhibited reduced H_2_O_2_ production [136]. The discrepancies between these studies may stem from variations in measurement approaches, cell types, and experimental conditions, necessitating further investigation on the effects of hypoxia on IVD cells.

#### 2.2.3. Glucose Starvation

Cigarette smoking impairs nutrient delivery to the disc by promoting vasoconstriction, ultimately contributing to glucose starvation. Glucose starvation is widely associated with increased ROS production from both mitochondria and the ER, and diminished antioxidant defense. It is known that mitochondria are primary sources of ROS generation [137]. An increase in mitochondrial O_2_^•−^ was observed in cells undergoing glucose starvation, which may be attributed to a decrease in mitochondrial membrane potential [138]. In contrast, ETC-deficient cells failed to exhibit an increase in O_2_^•−^ levels, leading to the absence of autophagy and mitochondrial dysfunction [139]. These findings suggest that glucose starvation induces ROS generation in mitochondria. Additionally, the ER-resident enzyme NOX4 exhibits increased activity during glucose starvation, further contributing to ROS generation [140]. Glucose starvation also reduces antioxidant capacity by impairing NADPH and GSH production via the pentose phosphate pathway and TCA cycle [141].

The AMP-activated protein kinase (AMPK) pathway plays a crucial role in cell survival under glucose starvation [142]. Low ATP levels activate the LKB1-AMPK pathway, maintaining NADPH homeostasis by promoting its generation through fatty acid oxidation and reducing its consumption in fatty acid synthesis [141]. However, elevated mitochondrial ROS levels activate the AMPK/mTOR pathway, inducing autophagy [139]. Beyond mitochondrial mechanisms, ER-associated pathways mediate glucose starvation responses through the ATF4-CHOP-PUMA and ATF4-CHOP-DR5 axes [143], as well as the PERK/eIF-2α/ATF4 pathway [144]. Additionally, intracellular signaling between the ER and mitochondria via the ROS/ERK2/Nur77/TPβ axis regulates ATP production and antioxidant defenses under glucose starvation [145]. However, further investigation in IVD cell populations is necessary to further elucidate these effects.

In summary, environmental risk factors contribute to excessive ROS production in IVD cells. However, the precise mechanisms through which excessive ROS alter cellular activities and influence cell fate remain unclear. In the following section, we review the effects of ROS-induced redox imbalance and oxidative stress on the organelle level, both in general and specifically in IVD cells, to provide valuable insights for future research.

## 3. Effect of Oxidative Stress on Organelle Redox Imbalance

The impact of ROS on ER stress and mitochondrial dysfunction has been extensively studied in various diseases such as cancer, whereas its role in musculoskeletal disorders, such as IDD, has received comparatively less attention [146]. However, as the significance of ROS as a signaling molecule has grown, interest in its effects on the IVD and its role in degenerative remodeling has grown as well. A large portion of this research has focused on mitochondria, likely due to its roles in bioenergetics and apoptotic signaling [5,147,148]. However, this section of this review will examine how ROS and oxidative stress regulate and disrupt ER and mitochondrial function while also summarizing existing research on IVD cells.

### 3.1. Endoplasmic Reticulum

The ER is the largest organelle in the cell and, in its healthy state, provides a surface for ribosomes to synthesize proteins, folds proteins, and stores Ca^2+^ [149,150,151,152]. Protein folding occurs properly when PDI and ERO1 pass electrons from unfolded proteins to oxygen, folding the proteins and forming H_2_O_2_ as a byproduct. Disulfide bonds in oxidized PDI accept electrons from thiols on unfolded protein, becoming reduced and thereby facilitating the formation of disulfide bonds in the protein. Oxidized ERO1 then accepts the electrons from reduced PDI, re-oxidizing it for future protein folding. Subsequently, oxygen accepts the electrons from reduced ERO1, resulting in the formation of H_2_O_2_ as a byproduct [151,153]. The ROS produced here can serve both as signaling molecules and as facilitators of oxidative protein folding though interaction with Prx4, which converts the ROS into H_2_O while creating disulfide bonds in unfolded proteins [154,155]. While H_2_O_2_ is reduced to H_2_O by Prx4, the Prx family can also oxidize Trx to reduce H_2_O_2_ into H_2_O. Oxidized Trx can then be reduced by TrxR and NADPH for future ROS elimination [24,156]. In addition to the Prx/Trx system, the GSH system can reduce H_2_O_2_ into H_2_O by oxidizing two GSH molecules, with GPx7 or GPx8 serving as a catalyst [157]. Oxidized GSH can then be reduced back to GSH through electron exchange with NADPH, with glutathione reductase acting as a catalyst [24], as described previously (Figure 2). Along with oxidative protein folding, another major source of ROS generation in the ER is NOX4, an enzyme located in the ER lumen. NOX4 utilizes O_2_ and NADPH to produce H_2_O_2_, primarily for signaling purposes [158,159]. One use for signaling ROS is the regulation of Ca^2+^ levels through sarco/endoplasmic reticulum calcium ATPase (SERCA) and inositol 1,4,5-trisphosphate receptor (IP_3_R). ROS can interact with SERCA to prevent Ca^2+^ from entering the ER or with IP_3_R to allow for the expulsion of Ca^2+^ from the ER [160,161]. Ryanodine receptors (RyRs) are also associated with the release of Ca^2+^ from the ER [162].

**Figure 4:**
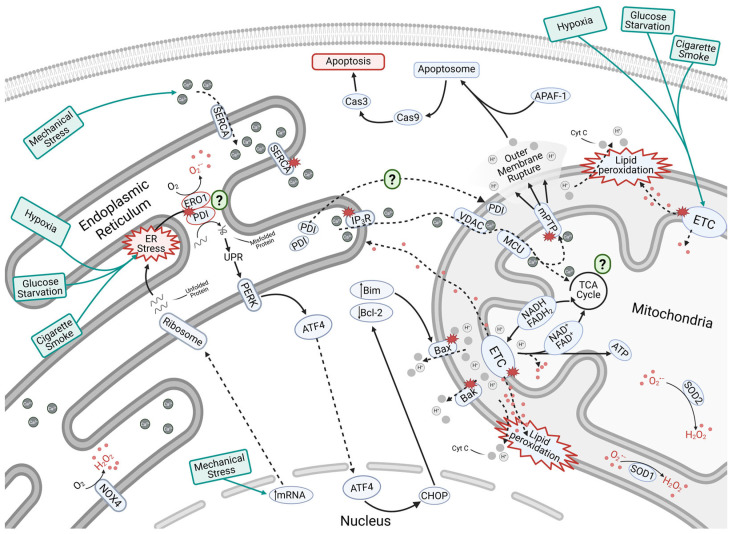
The cascade of ER stress and mitochondrial dysfunction along with their crosstalk. Environmental risk factors (cyan) directly and indirectly affect organelle function in IVD cells. Cigarette smoke, hypoxia, and glucose starvation can induce ER stress, resulting in hyperoxidation of protein disulfide isomerase (PDI) and increased endoplasmic reticulum oxidoreductin 1 (ERO1) activity, resulting in misfolded proteins triggering the unfolded protein response (UPR). Additionally, stress can result in the closing of sarco/endoplasmic reticulum calcium-ATPase (SERCA) channels and prolonged opening of inositol 1,4,5-trisphosphate receptor (IP_3_R) channels, altering Ca^2+^ homeostasis in the ER and triggering the UPR. Environmental risk factors can also affect the electron transport chain (ETC), resulting in increased ROS (red) generation initiating pro-apoptotic signaling cascades though either mitochondrial permeability transition pore (mPTP) or Bax/Bak permeabilizing the outer mitochondrial membrane. Crosstalk between the ER and mitochondria occurs at mitochondrial associated membranes where Ca^2+^ and ROS can be exchanged as inter-organelle signaling ions and molecules. ? indicate knowledge gaps in our understanding of IVD organelle function.

When cellular homeostasis is disrupted, ER stress can occur, manifesting as an accumulation of misfolded and unfolded proteins, increased ROS generation, disrupted Ca^2+^ homeostasis, and crosstalk with mitochondria (Figure 4) [163,164]. Oxidative stress can lead to the hyper-oxidation of PDI and increased H_2_O_2_ production by ERO1. Disruption of these two essential folding proteins results in accumulation of unfolded proteins, triggering the unfolded protein response [165]. The unfolded protein response activates signaling cascades aimed at restoring proper protein folding and preventing autophagy. However, when the accumulation of misfolded proteins becomes overwhelming and excessive oxidative stress is present, the PERK-eIF2α-ATF4 signaling cascade is activated. Protein kinase RNA-like endoplasmic reticulum kinase (PERK), an unfolded protein response sensor, is activated under oxidative stress, resulting in the phosphorylation of eIF2α in the cytosol [166]. This activation allows ATF4 to translocate to the nucleus, where it promotes CHOP-mediated upregulation of Bcl-2-Interacting Mediator of Cell Death (Bim) (pro-apoptotic protein) and downregulation of Bcl-2 (anti-apoptotic protein), leading to mitochondrial-induced apoptosis [167]. Additionally, the excessive ROS generated by increased mechanical stress and cigarette smoking can also induce ER stress, leading to the activation of this same pathway. While ER stress can indirectly contribute to ROS generation through ERO1-generated H_2_O_2_, it also appears to have a direct impact on ROS production through NOXs, specifically NOX4 in the ER [159]. The role of NOX4 in the ER remains debated; however, it is known to produce H_2_O_2_, which is believed to play a role in signaling within the ER and influence the unfolded protein response [159,168]. While protein load is a major source of ER stress, another key factor is the disruption of Ca^2+^ homeostasis. ROS can interact with active cysteines on IP_3_R, resulting in the expulsion of calcium from the ER to mitochondria [161]. Additionally, ROS can also modify SERCA receptors, inhibiting Ca^2+^ influx from cytoplasm to ER, further disrupting Ca^2+^ homeostasis [169,170].

IVD is a high-load environment for protein synthesis, requiring the ER to efficiently manage protein production and folding. Numerous studies have demonstrated that ER stress plays a crucial role in IDD [31,171]. The expression of ER stress-related markers, including GRP78, IRE1, ATF4, CHOP, and Cas-12, is significantly elevated in IDD [172,173,174,175,176]. Additionally, IVD degeneration is linked to increased oxidative stress in the ER. H_2_O_2_ promotes NP cell apoptosis via the ATF4/CHOP pathway [177,178], while peroxynitrite, a strong oxidant, leads to tyrosine nitrosylation, a marker of excessive ROS. Higher levels of nitrotyrosine-positive cells correlate with more severe IDD [179,180]. Oxidative stress and Ca^2+^ imbalance also contribute to IDD progression. The ER regulates intracellular Ca^2+^ storage [176], but prolonged ER dysfunction releases Ca^2+^ into mitochondria, triggering apoptosis [181]. The IP3R-GRP75-VDAC1 pathway regulates this process [176]. AGEs can elevate intracellular Ca^2+^, deplete ER Ca^2+^ stores, and induce NP cell apoptosis through ER stress, worsening IDD [32]. Future studies should focus on uncovering the mechanisms through which ROS regulate ER-mediated signaling within the harsh environment of the IVD.

### 3.2. Mitochondria

Another major organelle, the mitochondrion, also plays a critical role in redox signaling [182]. Historically, mitochondria were recognized for their role in ATP generation through oxidative phosphorylation. However, they are now also known to play a crucial role in signaling pathways, including apoptotic signaling, which is mediated by mitochondrial ROS and its crosstalk with the ER. Within the mitochondrial matrix and along the inter-mitochondrial membrane, the TCA cycle and ETC interact to produce energy for the cell through oxidative phosphorylation [183,184]. The TCA cycle produces NADH and FADH_2_ as byproducts, which are subsequently utilized in ETC. The ETC accepts electrons from these two molecules, converting them back into NAD^+^ and FAD^+^, which can then return to the TCA cycle for reuse. The electrons accepted during this reaction are used by the four ETC complexes to pump hydrogen ions (H^+^) from the matrix to the intermembrane space, creating a membrane potential for ATP generation. However, during this process, electrons can “leak”, predominantly from complexes I and III, resulting in the formation of O_2_^•−^ [24,185,186,187]. Under normal conditions, the generated O_2_^•−^ undergoes dismutation by SOD1 and SOD2 to form H_2_O_2_, which can then be neutralized by the mitochondrial specific Prx3 and GPx4 (Figure 4) [27,188]. Since mitochondria produce large quantities of ROS, Prx3 can become overoxidized when its free thiols (Prx-SH) are oxidized multiple times, becoming sulfinic acids (Prx-SO_2_H). However, Srx can reverse this overoxidation, restoring Prx3 function and ensuring continued antioxidant protection [27]. Srx has also been shown to act as a deglutathionylation enzyme and play a pivotal role in restoring redox homeostasis following oxidative damage [189,190].

When ROS production outpaces antioxidant protection in the mitochondria, oxidative stress becomes detrimental to the cell. This can result in mitochondrial DNA damage, lipid peroxidation, and ETC dysfunction (Figure 4). Under oxidative stress, the ETC experiences increased electron leakage, leading to elevated O_2_^•−^ generation. This excess ROS can trigger lipid peroxidation, damaging the outer mitochondrial membrane, resulting in membrane depolarization [191]. This impairs ATP production and facilitates the release of cytochrome C (Cyt C) into cytoplasm, initiating the apoptotic process [192]. Additionally, excess ROS can result in Bak/Bax translocation to the outer mitochondrial membrane, promoting membrane permeabilization. This again leads to release of Cyt C and H^+^ into the cytosol, contributing to membrane depolarization and apoptotic signaling [146]. Oxidative stress can also trigger the opening of the mitochondrial permeability transition pore (mPTP), a channel in the inner mitochondrial membrane that permits the free passage of large molecules (<1.5 kDa) from the mitochondrial matrix to the intermembrane space. In addition to oxidative stress, the mPTP can be activated by ATP depletion and excess Ca^2+^, leading to mitochondrial membrane swelling. If the pore remains open for a prolonged period, it can cause membrane rupture, ultimately triggering apoptosis [26,148,193].

Much more research has been conducted on the effect of ROS on mitochondrial signaling in IVD cell populations, likely because mitochondria are considered the primary generator of ROS within the cell [24]. Research on mitochondrial ROS signaling and oxidative stress can be broadly categorized into two somewhat overlapping areas: bioenergetics and apoptotic signaling. The following sections will explore these two topics in detail.

#### 3.2.1. Mitochondrial Bioenergetics

An accessible way to examine IVD mitochondrial bioenergetics is by measuring membrane potential and ATP levels in cell populations. Mitochondrial membrane potential describes the electrical potential gradient present across the inner mitochondrial membrane, which is generated by the ETC as it pumps H^+^ from the mitochondrial matrix to the intermembrane space. A high membrane potential indicates healthy mitochondria capable of producing energy. Din et al. investigated the effects of compression on rabbit NP monolayer cell cultures and found that with increasing compression duration, the ROS levels increased and membrane potential decreased [194]. The study postulated that ROS might play a role in the decline in membrane potential [194]. Additional studies that introduced H_2_O_2_ or TNF-α to human or rat NP cell cultures confirmed this finding, observing a decrease in membrane potential with increased exposure time [195,196,197]. Since membrane potential drives ATP production via ETC, a decline in membrane potential leads to a reduction in ATP levels. Studies examining the effects of compression-induced mitochondrial dysfunction in human and rat NP cells found that compression not only increased ROS generation within mitochondria but also decreased the ATP levels [198,199]. This insinuates that mitochondrial-derived ROS play a role in the alteration in mitochondrial bioenergetics, leading to a decrease in mitochondrial membrane potential and ATP production [194,198]. While these studies show the effect of ROS on NP cells, it must be noted that culture conditions were not physiologically representative of native NP tissue. The NP cell cultures were maintained under normoxic conditions (21% O_2_) with high glucose concentrations, which differ from the hypoxic, nutrient-limited environment of the native NP. Nasto et al. showed that culturing human NP cells at 20% O_2_ led to increased ROS generation compared to a more physiological 5% O_2_ environment [182]. Normoxic culture resulted in increased O_2_^•−^ concentrations, which are associated with mitochondrial respiration. Culturing NP cells in this normoxic environment likely enhances oxidative phosphorylation in mitochondria, resulting in greater electron leakage from ETC and subsequent ROS generation. The addition of compression loading or H_2_O_2_ may further exacerbate mitochondrial bioenergetic deterioration.

#### 3.2.2. Mitochondrial-Mediated Apoptosis

In addition to energy production, recent studies have shown that mitochondria also play a key role in apoptosis via ROS-induced signaling. As previously mentioned, mitochondrial-mediated apoptosis occurs either though prolonged opening of the mPTP, or Bax/Bak-mediated permeabilization of the outer mitochondrial membrane, both resulting in Cyt C release into the cytosol. Cyt C release then initiates the formations of apoptosomes, leading to the activation of Cas-3 and Cas-9, ultimately driving apoptotic cell death. Previous studies have shown compressive-testing-induced ROS generation in rabbit, rat, and human NP cell cultures, along with increased opening of the mPTP [194,198,199]. While these studies do not directly demonstrate that ROS triggers mPTP opening, they suggest the two are associated. Additionally, following compression, both Cas-3 and Cas-9 were upregulated, further linking mPTP opening to mitochondrial mediated-apoptosis in the IVD [194,198,199].

The Bax-mediated outer mitochondrial membrane permeabilization has been examined as an alternative pathway for ROS-induced mitochondrial-mediated apoptosis. Typically, Bax is found in the cytoplasm in an inactive state, bound to Bcl-2. However, decreased Bcl-2 expression can result in Bax translocation to the outer mitochondrial membrane, where it induces membrane permeabilization and subsequent release of Cyt C, initiating the apoptotic cascade. To investigate this pathway, Bax and Bcl-2 expressions have been measured in various studies on NP cell populations. Studies examining compression-induced apoptosis have reported increased Bax and Cas-3 mRNA and protein expression, as well as decreased Bcl-2 mRNA and protein expression, alongside increased ROS generation in human and rabbit NP cells [194,199]. Furthermore, studies using H_2_O_2_ or inflammatory factors to induce oxidative stress in rat and human NP cells showed increased Bax and decreased Bcl-2 expression, along with elevated cytosolic Cyt C and cleaved Cas-3 expression [195,196,197]. These studies, combined with the compression studies on NP cells, show that increased ROS exposure in NP cell populations results in activation of the Bax-mediated mitochondrial apoptotic signaling pathway.

### 3.3. ER–Mitochondria Crosstalk

While ROS can affect both the ER and mitochondria separately, recent research has shown that alterations in either organelle can trigger crosstalk between them [200,201] (Figure 4). While these are distinct organelles, they physically interact, and form contact sites through the formation of mitochondria-associated ER membranes (MAMs) in cholesterol-rich microdomains. MAMs are crucial for both Ca^2+^ and lipid metabolism. For example, ROS-induced Ca^2+^ release from the ER can lead to its uptake by mitochondria via the voltage-dependent anion channel (VDAC) on outer mitochondrial membrane and mitochondrial calcium uniporter (MCU) on inner mitochondrial membrane [160,202]. Increasing mitochondrial Ca^2+^ levels can then lead to the opening of the mPTP. As previously discussed, mPTP opening can result in Cyt C release, ultimately triggering mitochondrial-mediated apoptosis [150,201,203]. Additionally, Ca^2+^ can enhance ATP production through increased TCA cycling and subsequent ETC activity [183]. This can lead to increased ROS generation, particularly from complexes I and III of ETC. Additionally, indirect crosstalk between the ER and mitochondria can occur through signaling cascades that originate in one organelle and influence the other. As previously mentioned, oxidative stress activates the PERK-eIF2α-ATF4 pathway during the unfolded protein response in the ER, leading to increased Bim protein in the cytoplasm [167]. Bim facilitates Bax/Bak mitochondrial insertion into the outer mitochondrial membrane, leading to membrane permeabilization, Cyt C release, and apoptosis [146,204].

In contrast to the understanding of ER–mitochondria crosstalk in other tissue types, little is known about this interaction in IVD cell populations. Zhao et al., using an in vivo rat model, found increased apoptosis, GRP78 expression, and Cyt C expression after IDD was induced through surgical means [205]. These findings showed that upregulation of the unfolded protein response in the ER coincided with the release of Cyt C from mitochondria, leading to cellular apoptosis. In vitro experiments on rat AF cells demonstrated that introducing NS3694, an inhibitor of apoptosome formation, reduced apoptosis, while GRP78 remained upregulated [205]. This indicates that ER stress alone did not cause significant apoptosis in their model. Instead, crosstalk between the ER and mitochondria was dominant in initiating apoptotic signaling. Additionally, Lin et al. used rat NP cells to examine the effect of compression on ROS-mediated crosstalk between the ER and mitochondria [176]. This work found that compression leads to upregulation of CHOP, GRP78 and PERK, proteins associated with the unfolded protein response, as well as mitochondrial Ca^2+^ levels. By selectively inhibiting the IP_3_R and RyR ion channels in the ER at the MAM, it was discovered that IP_3_R was responsible for Ca^2+^ efflux from the ER to mitochondria in response to compression-induced ER stress [176]. This, in turn, could lead to mitochondrial Ca^2+^ overload and opening of the mPTP, triggering apoptosis, as has been shown in murine podocytes [203].

While the sections above summarize the current knowledge on apoptotic pathways related to the ER and mitochondria, further investigation on the effects of ROS on ER function and ER–mitochondrial crosstalk is needed to elucidate the progression of IDD. Specifically, regarding the ER, studies examining the effect of ROS on PDI and ERO1 should be conducted as these proteins are essential in protein folding and can lead to activation of the unfolded protein response in other tissues. Additionally, the ROS producer NOX4 and antioxidant systems GSH and Prx should be investigated, as they contribute to maintaining the oxidizing environment of the ER. Finally, most current studies focus on NP cell populations; however, the AF and CEP cells populate tissues with different functions that experience unique loadings and nutrient environments. This necessitates further investigation into the effect of ROS at the organelle level in IVD cell populations.

### 3.4. Oxidative Stress Biomarkers

While understanding the pathways through which oxidative stress and ROS induce apoptosis provides insights into current targets for treatment of IDD, standards through which oxidative stress can be quantified are necessary for early detection of IDD and intervention. Biomarkers and diagnosis tools can be used in various ways to determine the severity of IDD. First, global biomarkers could be used to examine the effect environmental factors have on IVD health. The environmental factors mentioned in this review act in a nonspecific manner. As previously mentioned, increased ROS can result in lipid peroxidation (Figure 4). Malondialdehyde is a byproduct of this process and has been measured in body fluids of smokers and non-smokers, showing some potential for use as a biomarker [206,207]. It has also been measured in conjunction with increased ROS in vitro when human NP cells indicate increased lipid peroxidation under oxidative stress in the IVD [208]. Additionally, SOD expression in serum has been examined as a potential biomarker for oxidative stress in stroke-associated infections [209]. In either case, these biomarkers would only provide an overview of the effect ROS induced by any external stimuli was having on a patient.

Local biomarkers in the IVD are needed for direct early diagnosis of IDD. Measuring local biomarkers in the IVD is especially difficult as potential protein or gene expression, which are associated with disc degeneration and would act as good biomarkers, require samples from the IVD, samples which cannot be obtained without compromising the IVD structure. Pereira et al. examined potential biomarkers and diagnosis techniques, finding imaging techniques and subjective pain scales to be the most reliable method to diagnose IDD [210]. They found that MRI and MR elastography-derived stiffness were the most common methods used in imaging diagnoses. Using these diagnosis tools, however, IDD is diagnosed at a state in which surgical intervention is the likely outcome and degeneration has progressed beyond reversible remodeling. New imaging techniques need to be developed to specifically focus on the IVD and its relationship with redox homeostasis to provide an early diagnosis for IDD. One imaging technique that has shown promise in examining redox homeostasis in vivo is fluorescence lifetime imaging microscopy. This imaging technique is minimally invasive and utilizes the label-free autofluorescence of NADPH and FAD^+^ to analyze the redox state of brain tissue in vivo [211]. Dynamic glucose-enhanced MRI is another imaging technique that has been used in the brain to measure glucose concentrations in vivo. This imaging technique could be beneficial again in determining the metabolic activity of a tissue to gain an understanding of its redox state [212]. Moreover, IDD has been associated with the endplate calcification and ossification in vivo [17]. High-resolution CT imaging holds significant potential for clinical application in diagnosing the stage of IDD by enabling a detailed assessment of spatial calcification patterns and morphological characteristics of the IVD, providing insights into disease progression. Adapting these technologies for use in musculoskeletal tissues such as the IVD could enable the detection of early-stage IDD, allowing for timely clinical interventions.

## 4. Therapeutic Implications

While surgical interventions such as spinal fusion, discectomy, and artificial disc replacement remain the standard treatments for advanced stages of IDD [213,214], a range of molecular therapies and emerging strategies have shown clinical potential for early-stage intervention. These approaches, including targeting antioxidant defense enhancement, organelle function restoration, and ROS scavenging, offer advantages such as minimal invasiveness, preservation of native disc structure and motion, and reduced cost and complication rates. This section examines three therapeutic approaches: activating mechanotransduction pathways to boost antioxidant defense, mitigating ER stress and mitochondrial dysfunction, and introducing universal ROS scavenging to reduce ROS levels.

### 4.1. Targeting Mechanotransduction Pathways to Boost Antioxidant Defense

As discussed above, the activation of mechanotransduction signaling pathways can enhance antioxidant synthesis through Nrf2 activation. Various molecules can initiate signaling to reduce ROS levels and mitigate oxidative-stress-induced damage. The following therapeutic strategies focus on the PI3K/Akt pathway.

Naringin is a natural flavonoid glycoside known for its ability to activate antioxidant pathways, such as PI3K/Akt and Nrf2 [215,216]. Studies have shown that naringin significantly reduces intracellular H_2_O_2_-induced ROS levels by activating the PI3K/Akt pathway in cultured rat NP-derived mesenchymal stem cells (MSCs), as well as protect the NP-derived MSCs from apoptosis; however, this protective effect was attenuated when PI3K was inhibited [216]. These findings suggest that naringin enhances antioxidant defense via the PI3K/Akt pathway, potentially promoting Nrf2 activation and indicating its clinical potential for the treatment of IDD. Similarly, epigallocatechin gallate (EGCG), a bioactive polyphenol, has also been shown to activate antioxidant pathways. EGCG increases the survival of IVD cells in vitro exposed to H_2_O_2_-induced oxidative stress, whereas PI3K inhibition attenuates its antioxidant capacity [217], suggesting EGCG enhances antioxidant defense through the PI3K/Akt. Interestingly, no significant difference was observed between PI3K-inhibited and non-inhibited groups at 24 h, while a difference became evident at 48 h [217], indicating a delayed interaction between EGGG and the PI3K/Akt pathway. Stem cell therapy is a clinically promising approach that promotes self-healing [5]. One study has shown that CESC-derived exosomes (N-Exos) can reduce apoptotic proteins, such as Cas-3 and Bax, and protect cultured rat NP cells from apoptosis through activation of the PI3K/Akt pathway. However, PI3K inhibition effectively reversed this protective effect [218].

Overall, various molecules can activate mechanotransduction signaling pathways to reduce ROS levels by enhancing antioxidant expression, highlighting their clinical potential for the treatment of IDD. However, the mechanisms through which these molecules regulate antioxidants and redox homeostasis in IVD cells require further investigation.

### 4.2. Mitigating ER Stress and Mitochondrial Dysfunction

Another potential target for therapeutic intervention in IDD is addressing ER stress and mitochondrial dysfunction. Certain small molecules have demonstrated potential as stress-reducing agents by reducing ROS generation and protein misfolding [219]. 4-Phenylbutyric acid (4-PBA), an FDA-approved small molecule chaperone [220], demonstrated clinical potential in alleviating IDD [32,221,222]. In the presence of 4-PBA, cultured human NP cells stimulated with IL-1β and TNF-α show reduced expression levels of ER stress markers PERK and CHOP [221]. Similarly, 4-PBA has been shown to partially suppress cleaved Cas-3 and CHOP, which suggests that 4-PBA mitigates apoptosis in cultured human NP cells [32,222]. Tauroursodeoxycholic acid, an FDA-approved bile acid, has demonstrated its ability to protect rat NP cells in vitro from apoptosis under excessive mechanical compression by effectively reducing CHOP expression. This protective mechanism is thought to be mediated through the PERK and eIF-2α pathways [223]. Targeting or silencing molecules that inhibit unfolded protein response activation can reduce ROS generation and maintain Ca^2+^ homeostasis, thereby preventing excessive cell apoptosis and slowing IDD. GSK2606414, a PERK inhibitor, significantly suppressed the expression of TNF, ADAMTS5 transcription, and cleaved Cas-3 under starvation-induced ER stress in cultured human AF cells [224]. Moreover, silencing PERK or IRE1 expressions increased the synthesis of collagen II and aggrecan in human NP cells in vitro compared to the group treated with IL-1β and TNF-α. However, silencing ATF6 did not restore their protein expression levels [221]. These findings suggest that not all unfolded protein response pathways contribute to IDD, and further investigation is required to clarify their specific mechanisms for potential therapeutic strategies.

Mitoquinone is a mitochondrial-targeted antioxidant with clinical potential that mitigates ROS by selectively accumulating on the inner mitochondrial membrane [225]. A study reported that mitoquinone treatment in cultured human NP cells reduced compression-induced ROS generation and apoptosis [226]. Mechanistically, mitoquinone restored membrane potential and attenuated the opening of the mPTP, leading to reduced ROS production [226]. Additionally, it regulated mitochondrial fission–fusion dynamics, facilitating the removal of dysfunctional mitochondria and further lowering the ROS levels. Moreover, it enhanced autophagy through the PINK1/Parkin-mediated pathway, restored lysosomal protease activity, and maintained a low pH environment, preventing excessive ROS accumulation. Melatonin, an indole hormone [227], acts as a mitochondrial protector by regulating autophagy and mitophagy to maintain mitochondrial quality and reduce excessive ROS generation [228,229]. Specifically, melatonin promotes autophagy by inhibiting NF-κB activation in cultured human NP cells and activates Sirt1 in cultured rat endplate chondrocytes [228,229]. Additionally, melatonin stimulates Parkin-mediated mitophagy to clear dysfunctional mitochondria in rat NP cells in vitro, thereby reducing ROS levels and attenuating tert-butyl hydroperoxide induced cellular apoptosis [230]. Moreover, gene editing, particularly CRISPR/Cas9 technology, provides a promising clinical approach for directly modifying mitochondrial genes, such as replacing defective gene fragments with functional ones to restore mitochondrial function [5,231]. Stem cell therapy can also promote the restoration of mitochondrial structure and function [5,232]. Specifically, in cultured rat NP cells, MSC-derived exosomes reduce H_2_O_2_-induced mitochondrial ROS and may also supply mitochondrial proteins to aid in functional recovery [233].

### 4.3. Therapeutic Strategies for Introducing Universal ROS Scavengers

GSH, a major intracellular antioxidant, is essential for redox homeostasis and its alteration plays a key role in health and disease [234]. It directly neutralizes ROS. GSH serves as a cofactor for GPx, facilitating the detoxification of oxidants such as H_2_O_2_, as discussed in the previous chapter [235]. A study reported that 1 mM GSH not only effectively protected cultured human NP cells from H_2_O_2_-induced apoptosis but also significantly upregulated the gene expression of ECM components collagen II and aggrecan, which had been reduced by H_2_O_2_ exposure [236]. These findings highlight the clinical potential of GSH in IDD treatment. N-acetylcysteine (NAC), a precursor of GSH and potent scavenger of ROS [237], has been shown to inhibit H_2_O_2_-induced apoptosis and calcification of human CEP cells in vitro by downregulating the ROS/p38/ERK/p65 signaling pathway [238]. Additionally, NAC has been shown to preserve ECM integrity in TNF-α-exposed rat AF cells in vitro by reducing the gene expression of MMP-3 and COX-2, thereby preventing the loss of aggrecan [180]. Oral administration of NAC in a rat in vivo model alleviated puncture-induced IDD [180], indicating its potential clinical applicability in IDD treatment. Fullerols, with multiple hydroxyl groups, act as electron donors and acceptors, enabling them to effectively scavenge ROS [239]. In cultured mice vertebral bone marrow stromal cells and human NP cells, fullerols can suppress ROS levels induced by IL-1β and H_2_O_2_, resulting in increased anabolic gene expression of collagen II and aggrecan, and reduced catabolic gene and protein expression of MMP-3, MMP-9, MMP-13, and ADAMTS5 [240,241]. Resveratrol, a natural ROS scavenger, exerts its antioxidant effects through electron donation. One study demonstrated that resveratrol effectively scavenges intracellular ROS, protecting cultured rat NP cells from apoptosis induced by sodium nitroprusside [242]. This underscores resveratrol’s therapeutic potential for IDD.

## 5. Discussion and Future Perspectives

This review highlights the interplay between environmental risk factors and redox imbalance, which ultimately triggers a cascade of biological events in IVD cells. Numerous studies have explored how environmental risk factors contribute to ROS accumulation in IVD cells by introducing exogenous substances and modulating key signaling pathways [18,94,122,141]. ROS accumulation leads to reduced biosynthesis while promoting apoptosis, inflammation, and ECM degradation in IVD cells [72,243]. However, the precise mechanisms through which ROS-induced oxidative stress disrupts redox homeostasis, regulates cellular activities, and determines cell fate in the IVD—particularly through ER and mitochondrial pathways—remain unclear, especially under conditions of mechanical stress and smoking.

The ER plays a pivotal role in synthesizing and post-translationally modifying proteins through oxidative folding, making it a site of ROS generation [149,150,151,152]. Oxidative stress can induce ER stress, leading to the activation of the unfolded protein response and, while initially protective, prolonged activation becomes detrimental as the accumulation of misfolded proteins disrupts biosynthetic processes and reduces the production of critical ECM components [165]. Furthermore, severe ER stress along with prolonged unfolded protein response activation can trigger mitochondrial-mediated apoptosis, further compromising tissue homeostasis. Due to the limited research on ER stress in IVD cells, investigating the effects of ROS on PDI and ERO1 is particularly relevant, as they are critical for protein folding, which, if hindered, can lead to unfolded protein response activation. Additionally, further investigations into Ca^2+^ transport and storage are necessary due to their importance in intracellular signaling. Furthermore, the ROS producer NOX4 and antioxidant systems, GSH and Prx, should be explored as they are essential in maintaining the redox homeostasis in the ER [159,168,236]. Mitochondria, central to energy production and cell fate regulation, are equally vulnerable [182,183]. Compared to the ER, significantly more research has examined the effects of ROS on mitochondria in IVD cells, likely because mitochondria are considered the primary generators of ROS and are heavily involved in energy production [244]. Given the unique microenvironment of the IVD characterized by hypoxia and glucose starvation, the impact of excessive ROS on oxygen and glucose supply to the ETC and TCA cycle in mitochondria remains poorly understood and warrants further investigation. Importantly, ER stress and mitochondrial dysfunction are not isolated events but engage in bidirectional crosstalk; however, little is known about this crosstalk in IVD cell populations. Prior studies have suggested that ROS signaling and Ca^2+^ transport are both involved in signaling between the ER and mitochondria [176]. In addition to Ca^2+^-mediated signaling, recent studies suggest that PDI may be involved in this crosstalk as a member of the PDI family, P5, has been found in mitochondria [245].

Furthermore, the IVD contains multiple cell types, including notochordal-derived cells in the NP, fibroblasts and fibrochondrocytes in the AF, and chondrocytes in the CEP, each residing within distinct microenvironments [9,11,12]. NP cells experience a hypoxic, glucose-starved, and acidic environment, whereas AF cells are exposed to higher oxygen levels and better glucose availability. As the primary transport interface, the CEP provides the most favorable nutrient conditions compared to the NP and AF. Recent research into ROS generation and organelle function in IVD tissues has been performed almost exclusively in NP tissue at normoxic conditions with high glucose concentrations [176,196,198,242]. Given these regional differences, cellular responses to environmental risk factors such as mechanical stress and cigarette smoking are likely to vary, necessitating additional studies designed on a region-specific basis to better determine how the natural environment of the cell populations affect their intracellular processes under environmental stress conditions.

Environmental risk factors can initially disrupt redox homeostasis in IVD cells, leading to oxidative stress. In response, IVD cells initiate multiple defense mechanisms to neutralize the oxidative stress and restore redox balance. However, if oxidative stress persists, it can impair essential cellular functions. Sustained stress can trigger ER stress and mitochondrial dysfunction, which disrupt key processes such as protein synthesis, post-translational modifications, and ATP production. Additionally, elevated levels of ROS can cause DNA damage, lipid peroxidation, and protein oxidation, further compromising cellular function [26] and ultimately leading to cellular apoptosis. As cellular dysfunction progresses, over time, the disc microenvironment deteriorates and becomes characterized by chronic inflammation, ECM degradation, calcification, and the formation of fiber microfractures [33,171]. This worsening microenvironment can, in turn, further promote oxidative stress in resident cells, creating a vicious cycle of degeneration. Eventually, the advanced stages of IDD can lead to clinical symptoms such as chronic pain and functional disability, most commonly manifesting as low back pain. In addition to environmental stressors, internal risk factors, such as genetic and epigenetic influences, play a critical role in the progression of IVD degeneration. For example, Battié et al. confirmed a substantial genetic contribution to disc degeneration, with heritability estimates ranging from 29% to 61%, depending on the specific phenotype and lumbar level [246]. Moreover, specific single-nucleotide polymorphisms and gene mutations have been associated with IDD [247]. Furthermore, environmental factors can influence epigenetic mechanisms such as DNA methylation, histone modifications, and non-coding RNAs, all of which modulate gene expression without altering the DNA sequence [248]. These epigenetic changes may contribute to the dysregulation of IVD cell function and promote degenerative processes.

Aging is a complex biological process influenced by both environmental and internal risk factors. It is characterized by several hallmarks [19], including the accumulation of unfolded or misfolded proteins in the ER [249], reduced ATP generation in mitochondria [250], impairment of the antioxidant defense system [251,252], and increased DNA methylation in promoter regions [253]. These alterations contribute to intracellular ROS accumulation, leading to redox imbalance and oxidative stress, both of which are closely associated with degenerative remodeling of the IVD. However, the precise mechanisms through which aging modulates cell fate and accelerates IVD degeneration remain to be fully elucidated. We do know, however, that GSH levels decline in the aging process and have been linked with a variety of age-related diseases. This can be attributed to diminished ROS scavenging as well as impaired protective redox-mediated signaling where GSH serves as a critical co-factor, S-glutathionylation.

To the best of our knowledge, no clinical trials have been conducted to evaluate the use of antioxidants for alleviating IDD. This highlights the need for a comprehensive understanding of redox imbalance in IVD cells, crucial not only for elucidating the mechanisms underlying IDD but also for developing targeted therapeutic strategies. Addressing these knowledge gaps could pave the way for innovative treatments aimed at restoring cellular homeostasis and halting or even reversing IDD.

## Data Availability

No new data was created or analyzed in this study.

## Abbreviations

The following abbreviations are used in this manuscript:

4-PBA	4-Phenylbutyric Acid
AF	Annulus Fibrosus
Cas	Caspase
CEP	Cartilage Endplate
COX	Cyclooxygenase
Cyt C	Cytochrome C
ECM	Extracellular Matrix
EGCG	Epigallocatechin Gallate
ER	Endoplasmic Reticulum
ERO1	Endoplasmic Reticulum Oxidase 1
ETC	Electron Transport Chain
FADH_2_	Flavin Adenine Dinucleotide
GPx	Glutathione Peroxidase
GSH	Glutathione
H_2_O_2_	Hydrogen Peroxide
HIF-1	Hypoxia Inducible Factor-1
HO-1	Heme Oxygenase-1
IDD	Intervertebral Disc Degeneration
IL	Interleukin
IVD	Intervertebral Disc
MCU	Mitochondrial Calcium Uniporter
MMP	Matrix Metalloproteinase
mPTP	Mitochondrial Permeability Transition Pore
MSC	Mesenchymal Stem Cell
NAC	N-Acetylcysteine
NADPH	Nicotinamide Adenine Dinucleotide Phosphate
NOX	NADPH Oxidase
NP	Nucleus Pulposus
Nrf2	Nuclear Factor Erythroid 2
O_2_^•−^	Superoxide
OH•	Hydroxyl Radical
PDI	Protein Disulfide Isomerase
Prx	Peroxiredoxin
RNS	Reactive Nitrogen Species
ROS	Reactive Oxygen Species
SOD	Superoxide Dismutase
TCA	Tricarboxylic Acid
TNF	Tumor Necrosis Factor
Trx	Thioredoxin
TrxR	Thioredoxin Reductase
VDAC	Voltage-Dependent Anion Channel
